# Factors That Control the Chemistry of the LOV Domain Photocycle

**DOI:** 10.1371/journal.pone.0087074

**Published:** 2014-01-27

**Authors:** Josiah P. Zayner, Tobin R. Sosnick

**Affiliations:** 1 Department of Biochemistry and Molecular Biology, The University of Chicago, Chicago, Illinois, United States of America; 2 Institute for Biophysical Dynamics, The University of Chicago, Chicago, Illinois, United States of America; US Naval Reseach Laboratory, United States of America

## Abstract

Algae, plants, bacteria and fungi contain Light-Oxygen-Voltage (LOV) domains that function as blue light sensors to control cellular responses to light. All LOV domains contain a bound flavin chromophore that is reduced upon photon absorption and forms a reversible, metastable covalent bond with a nearby cysteine residue. In *Avena sativa* LOV2 (*As*LOV2), the photocycle is accompanied by an allosteric conformational change that activates the attached phototropin kinase in the full-length protein. Both the conformational change and formation of the cysteinyl-flavin adduct are stabilized by the reduction of the N5 atom in the flavin’s isoalloxazine ring. In this study, we perform a mutational analysis to investigate the requirements for LOV2 to photocycle. We mutated all the residues that interact with the chromophore isoalloxazine ring to inert functional groups but none could fully inhibit the photocycle except those to the active-site cysteine. However, electronegative side chains in the vicinity of the chromophore accelerate the N5 deprotonation and the return to the dark state. Mutations to the N414 and Q513 residues identify a potential water gate and H_2_O coordination sites. These residues affect the electronic nature of the chromophore and photocycle time by helping catalyze the N5 reduction leading to the completion of the photocycle. In addition, we demonstrate that dehydration leads to drastically slower photocycle times. Finally, to investigate the requirements of an active-site cysteine for photocycling, we moved the nearby cysteine to alternative locations and found that some variants can still photocycle. We propose a new model of the LOV domain photocycle that involves all of these components.

## Introduction

In response to blue light, algae and plants use LOV domain-containing phototropins to activate signaling cascades that end in phototropism or chloroplast rearrangement [1]–[3]. LOV domains contain 100-150 amino acids and are members of the PAS domain superfamily [4]. Many LOV domains are found as a part of larger proteins [5] whereas others can function as single domains [6].

Light activation occurs when the non-covalently bound flavin mononucleotide (FMN) or flavin adenine dinucleotide (FAD) chromophore absorbs a photon [7] that excites the chromophore into a singlet state. This state converts with high probability through an intersystem crossing into a triplet excited state [8]. In the triplet state, the flavin forms a metastable covalent bond between its C4a atom and the sulfur of the neighboring cysteine [8]. The N5 atom of the flavin is reduced, binding a hydrogen atom that is thought to come from the active site cysteine [8]–[10]. The resulting cysteine adduct spontaneously decays in seconds to hours depending on the local side chain environment [11]–[13]. The decay rate is controlled by the deprotonation of the N5 atom, returning the chromophore to its non-covalently bound dark state [14].

The photocycle of the flavin in LOV domains typically elicits a reversible conformational change [8], [15]–[18]. In *Avena sativa* LOV2 (*As*LOV2), the change involves the unfolding of the A’α and Jα helices [19], [20]. This conformational change has been shown to activate kinase activity in full-length phototropins [21]–[23].

The photocycle of LOV domains is highly tunable by a large number of factors [11], [13]. Mutations to residues near the chromophore can shorten the photocycle of *As*LOV2 from 80 s to 6 s or lengthen it to days [13], [19], [24], [25]. The known mechanisms that shorten the lifetime of the photocycle include increased solvent accessibility and modification of the electrostatic environment [11], [19], [24]. The photocycle can be lengthened by altering the packing around the active-site cysteine or through the removal of electrostatic interactions [13], [26].

Although these effects focus on the stabilization and removal of the covalent adduct, reversion to the dark state is likely limited by a base catalyzed deprotonation of the flavin. This step has been suggested to involve a highly conserved glutamine residue [14], [26]. However, mutations to this residue in *Arabadopsis thaliana* LOV2 (Q1029L [27]) and *As*LOV2 (Q513L [26] and Q513A [19]) slow but do not abolish the photocycle, with the Q513A photocycle time remaining within a factor of three of the wild-type value. The ability of the photocycle to remain intact with mutations to Q513 is perplexing because this is the only obvious side chain within the vicinity of the chromophore with the proper functional groups to catalyze the deprotonation process.

Recent studies have suggested that water molecules can enter the chromophore binding pocket [28] and directly participate in the photocycle chemistry [28]–[30]. This possibility would rationalize the maintenance of the photocycle upon mutation to residues such as Q513, by allowing a side chain independent mechanism to facilitate the proton transfer.

Here we investigate the deprotonation step and other chemical events of the *As*LOV2 photocycle using a battery of mutations designed to alter side chain interactions with the FMN and the surrounding water molecules. We found mutations near the chromophore that can alter photocycle times from 2 s to over 2000 s. The N414, F494 and Q513 triad are nonessential for photocycling but are necessary for the minute lifetime likely needed for biological function. The data can be explained with a model where the N414 position, located ∼ 10 Å away from the chromophore, controls water access and H_2_O coordination to alter the photocycle lifetime. Experiments on dehydrated protein further establish the role of H_2_O molecules in the photoadduct decay. In an effort to understand the requirements to build and use photoactive PAS domains, we also created new active-site cysteine residues that photocycle on similar timescales as the wild-type protein. From these results we develop a new model of photocycle function and provide new directions that can be followed for the tuning and creation of optogenetic tools.

## Materials and Methods

### Cloning, Expression and Purification

A clone of *A. sativa* phot1 LOV2 (404-560) with a His_6_-G*β*1 fusion was used. Mutations were made using the quickchange site directed mutagenesis strategy. All proteins were expressed in *Escherichia coli* BL21 (DE3) cells grown in M9 minimal medium supplemented with ^15^NH_4_Cl (1 g/L) at 37°C to an OD 600 nm of 0.6 and induced with 1 mM IPTG. Cultures were then incubated for ∼18 hours at 18°C and pelleted and frozen. Frozen pellets were resuspended in 50 mM Tris 100 mM NaCl and 0.01% SDS and cells were lysed using sonication and clarified with centrifugation at 10000*g* for 45 min. The proteins were then purified using metal affinity chromatography and exchanged into 50 mM Tris, 1 mM EDTA, 5 mM DTT, pH 8. The His_6_-G*β*1 tag was removed by incubating overnight at 20°C with His_6_-TEV protease. Metal affinity chromatography was then used to remove His_6_-G*β*1 and His_6_-TEV protease from the solution. The final protein contains residues GEF on the amino terminus and G on the carboxy terminus as cloning artifacts. Proteins were run on a Sephadex S100 size exclusion column (GE Healthcare) and if the UV-Vis 280/447 nm ratio differed greatly from ∼2.6-2.9, proteins were further purified using anion exchange chromatography.

### UV-Vis

UV-Vis spectra were acquired using an Olis HP 8452 Diode Array (Bogart, GA) with a 2 nm bandwidth and a 1 cm pathlength cuvette at 22°C. Kinetic traces were acquired after photosaturation. Samples were illuminated using a 40 W white LED (Model no: BT DWNLT A, TheLEDlight.Com) for 30 seconds and the absorbance at the λ_max_, usually 448 nm, was measured every 1-30 seconds depending on the photorecovery rate. The data were fit to a single exponential decay using Origin software (OriginLab). The percent of a population excited was calculated by linearly extrapolating a bleached minimum at 448 nm and comparing it to the dark maximum at 448. Salt was removed from protein solutions before drying. Approximately 100uL of 10uM protein in solution was placed on the outside of a cuvette and air dried in the dark.

## Results and Discussion

The photocycle of LOV domains can be measured by monitoring A_448_, the absorbance near the λ_max_, usually at 448 nm. Upon photoexcitation, the N5 atom is reduced and gains a proton, changing the electronic nature of the chromophore and bleaching the spectra, causing A_448_ to reach a minimal value. Unless otherwise noted, the photo-recovery can be fit with a single exponential having a time constant, τ_FMN_. At 22°C, τ_FMN_ 81±2 s in WT *As*LOV2 (phot1 residues 404-560).

### Side Chain Effects on the Photocycle

**Figure 1:**
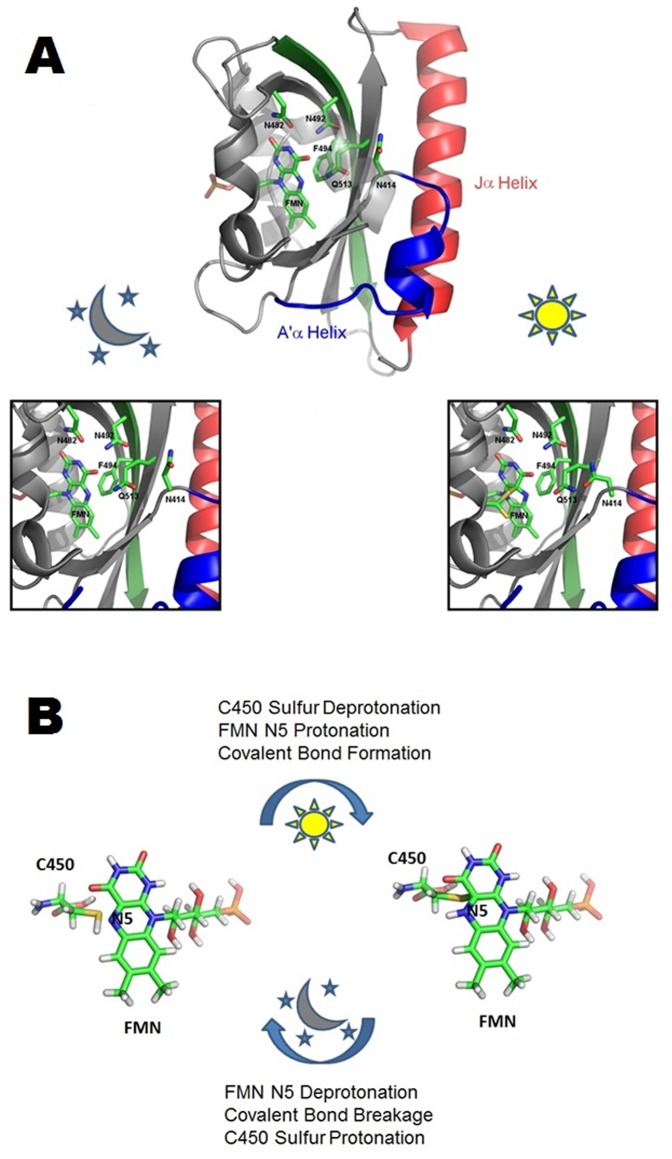
Effects on *As*LOV2 side chains during the photocycle. (A) Side chain positions in the chromophore binding pocket in the dark and light state crystal structures [32] (B) Chemical processes that occur to the FMN chromophore during the photocycle.

The LOV domain architecture provides an environment suitable for flavin molecules to undergo reversible reduction and oxidation [31]. A multitude of interactions between the flavin molecule and the protein exist inside the flavin binding pocket. In *As*LOV2, the flavin interacts electrostatically with the residues N482, N492 and Q513 and forms a π-bond or ring stacking interaction with F494 (Fig. 1A). Other residues near the chromophore have been shown to have effects on the photocycle but these generally are through indirect interactions such as increasing the solvent accessibility [11], [13]. Of these indirectly interacting residues, N414 is of note because of the large effects upon substitution [13], its conformational change between the light and dark crystal structures [4], [32] and its interactions with Q513 in simulations [4], [13], [30].

We used mutations to probe the residues suggested to regulate the photocycle of *As*LOV2, N414, N482, N492, F494 and Q513 (Fig. 1A). Except for N414, these residues are highly conserved in LOV domains with F494 occasionally being replaced with a leucine. Using UV-Vis spectroscopy, we measured how these mutations influence the lifetime and changes in λ_max_ (Table 1). Five of the variants (N482A, N492Q, F494N,W or Y) did not express in sufficient quantities to be characterized, as previously observed in other mutants [13], most likely because of compromised FMN binding.

**Table 1:** Chromophore and Photocycle parameters of *As*LOV2 variants.

Construct	τ_FMN_ (s)	λ_max_(nm)	Source
WT	80	448	Zayner *et al*. 2012
N414A	1427	448	This Work
N414D	69	446	This Work
N414G	615	448	This Work
N414L	1847	444	This Work
N414Q	280	448	This Work
N414S	685	448	Zayner *et al*. 2013
N414T	892	448	Zayner *et al*. 2013
C450V	NM	448	This Work
L453V	160	446	This Work
N492A	54	448	Zayner *et al*. 2012
F494L	206	448	Zayner *et al*. 2012
F494H	NM	444	This Work
Q513A	261	442	Zayner *et al*. 2012
Q513D	5	448	This Work
Q513H	30	446	This Work
Q513L	1793	438	This Work
N414A/Q513H	2	446	This Work
N414L/Q513A	1900	444	This Work
N414A/Q513A	2081	442	This Work
Dehydrated† WT	1726	448	This Work
Dehydrated† Q513A	860	442	This Work
Dehydrated†N414L/Q513A	4076	444	This Work
F494C	282	448	This Work
C450V/F494C	69	448	This Work
C450V/Q513C	44	440	This Work
C450V/L453C	NM	448	This Work
N414T/C450V/Q513C	NM	440	This Work
N414G/C450V/Q513C	13	440	This Work
WT +1mM Imid.	12	448	This Work
WT +2mM Imid.	6	448	This Work
WT +3mM Imid.	4	448	This Work
C450V/Q513C +1mMImid.	9	440	This Work
C450V/Q513C +2mMImid.	9	440	This Work
C450V/Q513C +3mMImid.	8	440	This Work

† Samples were air dried for 48 hours.

Other variants of N414, N492, F494 and Q513 typically lengthened the photocycle (Table 1). These variants disrupt interactions with the chromophore or change the steric packing near it, factors that have been previously shown to affect photocycle lifetime [11]. To further understand the influence of N414, F494 and Q513, we mutated them to a number of different functional groups. Based on our prior observation that N414V has 12+ hour long photocycle [19], we also mutated N414 to A, D, G, L, S, and T substitutions. All except N414D slow the photocycle by at least five-fold (Table 1). Substitutions at the N414 position have the most dramatic effect on the photocycle of any site we have investigated (other than the C450) [13], [19].

**Figure 2:**
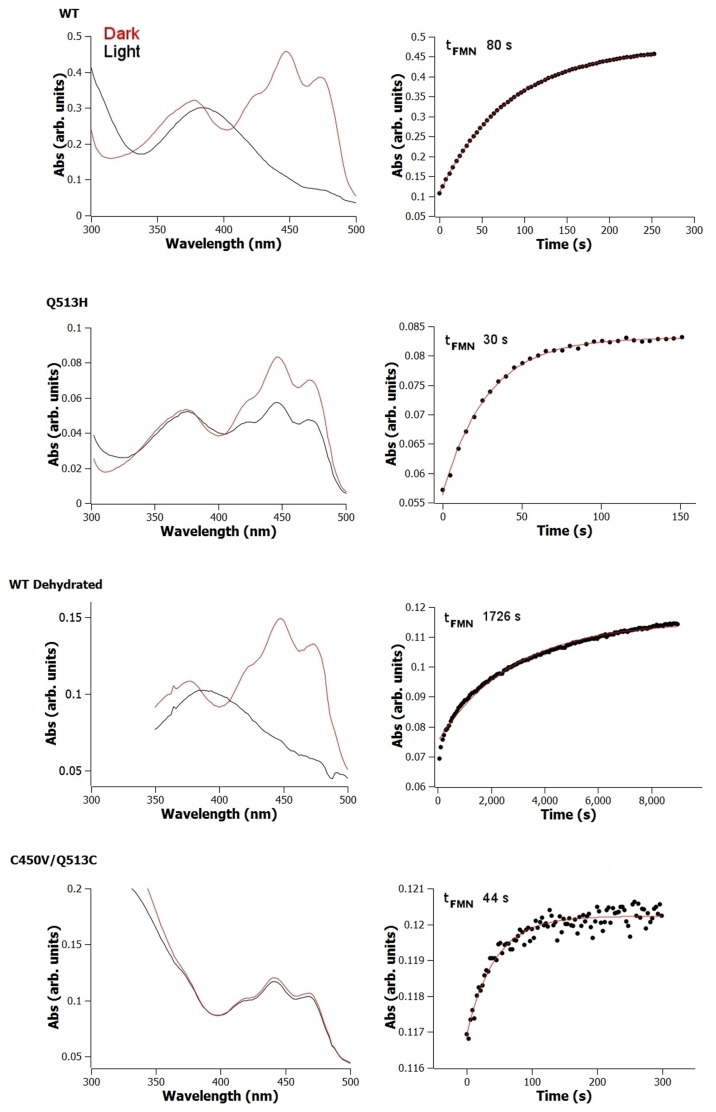
UV-Vis absorbance changes upon photoexcitation and recovery for selected *As*LOV2 variants. Single exponential fits to the time dependent data are shown in red.

Based on its location in the structure, the N414 residue may control water entry into the chromophore binding pocket (Fig. 1A). Crystal structures in the lit and dark state indicate rotations of N414 and Q513 during light activation (Fig. 1A) [32]. Likewise, the effects of N414 substitutions on τ_FMN_ relate both to residue size and electrostatic properties with larger hydrophobic side chains having the longer times as compared to smaller residues (e.g., N414L: τ_FMN_  = 1847 s>N414A: τ_FMN_  = 1427 s>N414G: τ_FMN_  = 615 s), polar residues have faster times (N414Q: τ_FMN_  = 280 s and WT N414: τ_FMN_  = 80 s) and a negatively charged residue has the shortest photocycle (N414D: τ_FMN_  = 69 s) (Table 1 and Fig. 2A). Notably, the alanine and glycine side chains still have slower photocycle times than the original asparagine in spite of their smaller size. These results at N414 indicate that sterics are not the overriding factor controlling life-time of the activated state at this position. Therefore, based on polar and charged residues in the N414 position having faster photocycle times and hydrophobic or neutral side chains having slower photocycle times, we suggest that N414 is involved in the coordination of the water molecules to catalyze a faster transfer of the proton.

A number of N414 mutations modify the electronic properties of the chromophore causing blue shifts in the λ_max_. Because the distance of N414 to the chromophore is ∼ 10 Å, it is unlikely that there is a direct effect. Rather, the shift is likely caused by a neighboring residue or by changes in the FMN’s solvation mediated by N414. Further, the N414’s influence on the FMN allows for a possible signaling mechanism from the chromophore to the A’α helix (located near N414), which has been shown to regulate light activated conformational change [19], [30].

Accordingly, we investigated whether the effect N414 has on the chromophore’s λ_max_ and photocycle time occurs independently or through a neighboring residue. The Q513 residue is positioned between N414 and the chromophore. Mutations to Q513 also shift the λ_max_ and change the τ_FMN_. We hypothesized that if the effect of N414 on the chromophore is through Q513 then by mutating Q513 to an inert functional group, mutations to N414 should have less or no effect on the chromophore. If N414 is acting directly on the FMN, however, we would expect effects even if the Q513 position is inert. For the N414L/Q513A variant, λ_max_  = 444 nm, the same as for the N414L mutant rather than the Q513A variant where λ_max_  = 442 nm. Therefore, N414L can act in a dominant fashion. The N414A mutant, however, does not shift λ_max_ but N414A/Q513A mutant shifts λ_max_ to 442 nm, which suggests a Q513 dominant or combinatorial effect for this pair (Table 1).

We observe similar behavior for the photocycle time. The N414L and N414L/Q513A have similar τ_FMN_, 1847 and 1900 s, respectively. However, an additive or synergistic effect is observed for the N414A/Q513A variant; its lifetime, 2048 s, is longer than either of single mutations (N414A: τ_FMN_  = 1427 s, Q513A: τ_FMN_  = 267 s; Table 1).

The N414 mutations in combination with the Q513 mutations suggest that the N414 affects the chemistry of the photocycle in a manner distinct from Q513. The N414L mutation is functionally dominant over the Q513A mutation in regards to photocycle time and λ_max,_ whereas the N414A and Q513A mutations appear additive. We believe that the N414 residue in *As*LOV2 influences solvent access or H_2_O coordination with the chromophore thereby regulating the chemistry of the photocycle.

These and other mutations to Q513 confirm that this residue has significant effects on photocycle lifetimes and the electronic nature of the chromophore, as measured by λ_max_ [13], [26], [27]. With an acidic substitution, Q513D, the protein has a much faster photocycle, 5 s, than most WT phototropin LOV2 domains, which typically range from 56 to 786 s [12] (Table 1). In contrast, hydrophobic residue substitutions slow down the photocycle by 20-fold (Q513L) and 4-fold (Q513A). The mutation of Q513H, intended to place a basic group near the FMN protonation site, has a photocycle time of 27 s, which is one third of the WT lifetime (Table 1). However, Q513H has the peculiar effect of not fully photobleaching even upon saturating illumination. For Q513H, the A_448_ is 43% of the WT value under the same 40 W illumination level (Fig. 2B). The mechanism of Q513H’s reduced photobleaching is unclear, potentially involving another species with a slow recovery time or altered spectral properties.

Changes in electrophilicity near the chromophore have been suggested to control the rate of intersystem crossing of the singlet to triplet state [8] but little is known about how electrophilicity effects the lifetime of the adduct state. Overall, we see an effect at the Q513 position that correlates with the electronegativity of the side chain. Side chains with protonated nitrogens that are thought to interact directly the chromophore (Q513, τ_FMN_  = 80 s and Q513N, τ_FMN_  = 43 s) have the slowest recovery times, whereas a residue with a neutral pKa (Q513H) has a slightly faster time, 27 s, and very electronegative functional groups (Q513D) photocycle in only 5 s (Table 1). Although Q513 effects the photocycle time, this residue does not seem to have any requisite role in the reversibility of the process; potentially, its major role is to propagate conformational change as seen in other studies [26], [27].

Previously, we have made the F494L mutation in *As*LOV2 [19] while the corresponding Phe to Leu mutation was introduced in the neo1 LOV2 domain [33]. In both organisms, the photocycle time is slowed 2-3 fold and there is a concomitant decrease in the amount of light activated conformational change [19], [33]. Many mutations at the F494 position failed to express though most were to large or charged residues (Asn, Trp, Tyr). With F494H, the photocycle completely vanishes under our illumination conditions with no evidence of significant flavin reduction measured similar to the Q513H mutation. We do not have an explanation for the profound effect that these histidines have on the photocycle.

Overall we can modify photocycle times and characteristics by affecting solvent accessibility through N414 mutations or the electrostatic environment of the chromophore. Non-cysteine side-chains near the chromophore in *As*LOV2, when mutated to inert functional groups, do not eliminate the photocycle. We conclude that these non-cysteine side-chains are not essential for the major chemical events (e.g., acting as a proton donor or acceptor) that permit the reversible process of the photocycle in LOV domains.

### Engineering of the Active Site Cysteine and the Effects of Imidazole

To determine the chemical properties that would allow the cysteine to be involved in proton donation and acceptance, and adduct formation, we performed a variety of measurements. We first attempted exogenous covalent bond formation with methyl mercaptan in a C450G mutant with experimental procedures the same as previously performed with *Chlamydomonas reinhardtii* LOV1 [34]. However, we could not reproduce this rescue experiment with *As*LOV2, potentially due to sequence and structural differences in the proteins.

**Figure 3:**
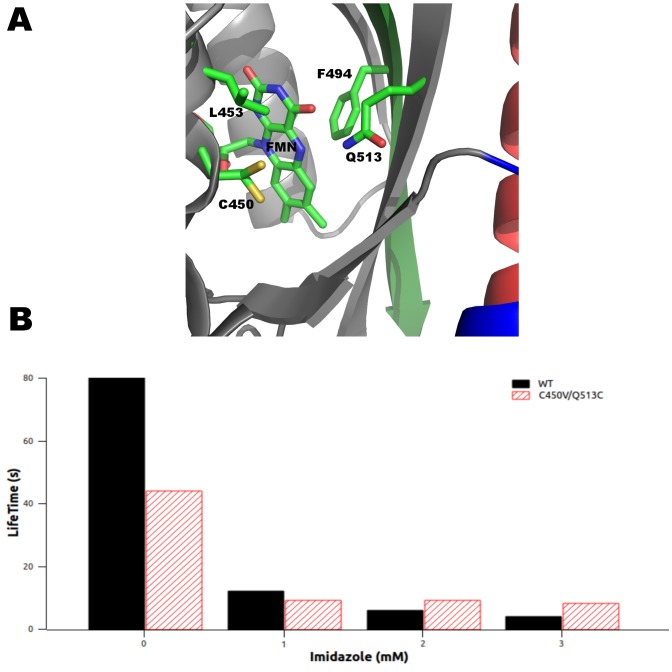
*As*LOV2 structure and side chains near the chromophore. (A) Location of side chains mutated in active-site cysteine variants (B) The effects of imidazole on photocycle lifetime in WT and C450V/Q513C.

The location and geometry of the active-site cysteine in relation to the chromophore has been suggested to affect the chemistry and duration of the photocycle [11]. However, we are unaware of any studies that have attempted to change the location of this critical residue. We moved the original cysteine (C450V or C450A) to three different locations near the chromophore based on distance and geometry constraints, L453, F494 and Q513 (C450A/L453C, C450V/F494C and C450V/Q513C) (Fig. 3A). The C450A/L453C variant lacked a measurable photocycle. But, the other two mutations C450V/F494C (τ_FMN_  = 69 s) and C450V/Q513C (τ_FMN_  = 44 s) had photocycle times very similar to WT (τ_FMN_  = 80 s) (Table 1). However only a small fraction of the population became photoexcited as indicated by minimal bleaching, <10%, (Fig. 2D and Fig. 3A).

We examined the effect of imidazole on C450V/Q513C variant’s photocycle. In the WT protein, the addition of imidazole hastens the photocycle [11], [14], with 1, 2 and 3 mM imidazole reducing τ_FMN_ from 80 s to 12, 6 and 4 s respectively (Table 1 and Fig. 3B). The C450V/Q513C variant also has a noticeable imidazole effect with τ_FMN_ shortened from 44 s to ∼9 s in 1-3 mM imidazole, very similar to the changes observed for the WT protein under the same imidazole concentrations (Table 1 and Fig. 3B).

We created a variant with altered solvent accessibility by introducing the N414G and N414T mutations in a C450V/Q513C background. We anticipated observing a change in photocycle times as was found with other variants when these mutations were made. The N414T/C450V/Q513C variant did not have a measurable photocycle, being too fast or non-existent. But the N414G/C450V/Q513C had a decrease in τ_FMN_ to 14 s compared to the 44 s for C450V/Q513C. The decrease may be due to structural changes arising from the multiple mutations causing a higher catalytic rate for water when the N414 group is absent (Table 1). The reversibility of the photocycle was unaffected by the new cysteine residues. However, the ability of the protein to form the covalent adduct or initially reduce the FMN is greatly decreased indicating that geometry plays a role in successful photoactivation.

### Hydration

In addition to cysteine location, interaction with solvent plays a role in the chemistry of photo-reversibility. Both experimental and molecular dynamics simulation studies suggest that solvent can enter the FMN binding pocket [28], [29] and accessibility increases the speed of the photocycle [11], [14]. Recently, a cluster of water molecules was implicated in deprotonation [29]. Experimentally, this property has been measured indirectly by inferring solvent accessibility from changes in isotope effects or a proposed base catalyzed imidazole reduction of the chromophore [11], [14]. Here we wanted to test directly the effect of water on the speed of the photocycle. To do so, we air dried three different variants (WT, Q513A and N414L/Q513A) of the *As*LOV2 domain for >48 hours in the dark on the outside of quartz cuvettes at 22°C, and measured the photocycle. We chose both Q513A and N414L/Q513A variants to remove the possibility that either the N414 or Q513 residues were functioning in place of water to facilitate reduction of the chromophore. For the dehydrated versions, the FMN molecule retains its characteristic spectra and the λ_max_ does not measurably shift from the solvated version (Table 1 and Fig. 2C). The photocycle, however, is slowed with τ_FMN_ decreasing by ∼2-20 fold (Table 1 **)**. The dehydrated variants lose their ability to undergo conformational change as measured by CD and by FTIR [35]. These results suggest that water plays a significant role in the decay of the photocycle and conformational change. The dehydrated proteins can be rehydrated and they regain the ability to undergo conformational change. This recovery is indicative of a lack of protein denaturation or disruptive conformational changes as we have only observed minimal refolding of *As*LOV2 after denaturation using guanidine hydrochloride or heat (data not shown).

Upon the removal of bulk solvent, we expected, but did not observe a change in the electronic environment around the chromophore sufficient to measurably shift λ_max_. Presumably, either the protein has tightly bound water molecules or the role of solvent in deprotonation is so transient that the effects are not easily measurable. The crystal structures of *As*LOV2 [32] do not contain bound H_2_O molecules near the chromophore. However, in NifL, a very similar PAS domain, such molecules are observed near the chromophore and are required for oxidation of its bound flavin [36]. Together, this suggests that coordination of bound water molecules could be a conserved mechanism of PAS domain flavin redox reactions.

## Conclusion

Over a hundred mutations have been performed on *As*LOV2 by us and others [11], [13], [19], [24], [26] yet the only residue required for photocycling is C450. These experiments suggest that the minimal requirements for a reversible photocycle are an active-site cysteine residue, a protein cage to protect the chromophore from solvent, and water to participate in the oxidation of the FMN [29]. The engineered cysteine variants provide new insight into the chemical process of how the covalent bond is formed with the flavin upon light excitation. Interestingly, the photocycle length in these modified cysteine variants are similar to the WT protein indicating that this geometry is not a critical factor for the deprotonation process.

During the photocycle of *As*LOV2, the FMN chromophore is reduced to FMNH and is spontaneously oxidized back to its ground state at room temperature. At the start of the photocycle, the cysteine loses a proton and is the logical donor to the FMN based on proximity [9], [37]. The other major candidate to be involved in deprotonation would be the Q513 residue but a number of studies [13], [26], [27] have shown that even upon mutation to inert functional groups LOV domains still photocycle with similar time constants. These data suggest that the deprotonation process only requires a cysteine and solvent. However, both Q513 and N414 affect the length of the photocycle and the electronic nature of the chromophore as seen in λ_max_ changes.

Recent molecular dynamics studies suggest that Q513 plays a role in conformational change and does not flip to place its oxygen near the N5 of the FMNH molecule or form stable hydrogen bonds with the N5 [30]. We likewise suggest that Q513 rotates upwards away from the FMNH and repositions N414 to increase solvent accessibility to the FMN (Fig. 3). These residues could coordinate water molecules causing the electronegativity of the solvent oxygen to destabilize the FMNH state, promoting the breakage of the covalent bond and the return of the proton to the cysteine, similar to observations on the NifL PAS protein [36]. This model is different than what is inferred by the lit and dark state crystal structures [32] where the Q513 side chain undergoes a rotation to form a hydrogen bond with the newly protonated N5 atom on the FMN.

**Figure 4:**
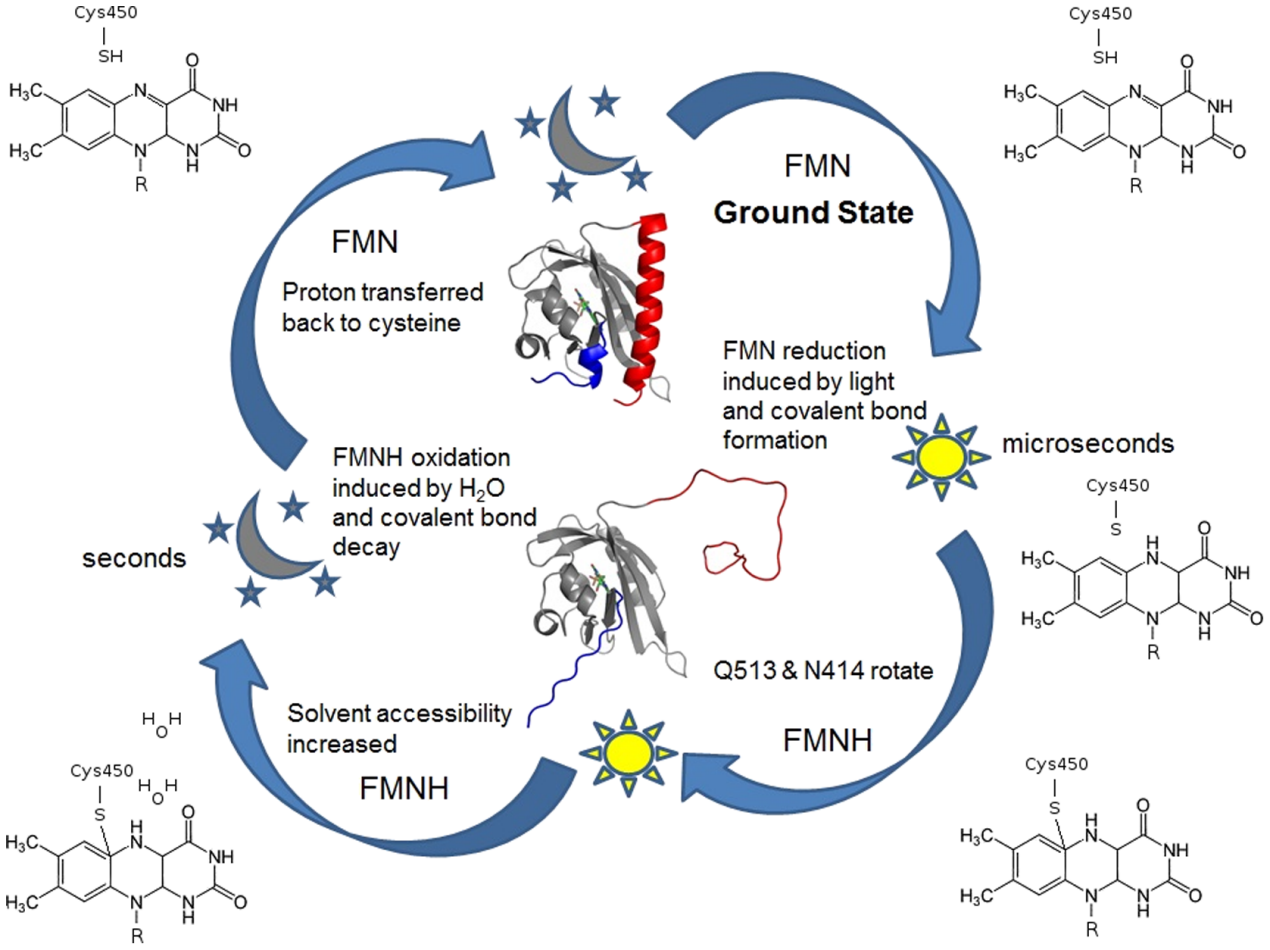
*As*LOV2 photocycle chemistry. Photon absorption results in the FMN being excited into a singlet then triplet state. The N5 of FMN becomes a strong nucleophile and removes the proton from the nearby C450 to form a covalent bond with the FMN C4a atom. Reduction of the FMNH state is inhibited by the inability of water to readily enter the chromophore binding pocket and catalyze the transfer of the proton back to the cysteine until a conformational change occurs involving residues N414 and Q513. The covalent bond is broken and the protein returns to the dark state.

Nevertheless, our experiments do indicate that N414 and Q513 influence the solvent accessibility and perhaps coordination with the FMNH. Though N414 is not a conserved residue in LOV domains such as Vvd and YtvA, they have much slower photocycle times, 18,000 and 3600 s, respectively, when compared to *As*LOV2 (80 s). This difference is further support that N414 affects solvent accessibility and controls H_2_O coordination, which would increase the catalytic rate of the proton leaving the FMNH. Though there are difficulties in measuring whether LOV domains require water for photocycling, we have determined that in air-dried conditions, the deprotonation step in the photocycle takes longer than in hydrated *As*LOV2. These results are synthesized in a model we created that provides a new framework to view how LOV domains can reversible photocycle (Fig. 4).
